# The Influence of Home-Court Advantage in Elite Basketball: A Systematic Review

**DOI:** 10.3390/jfmk9040192

**Published:** 2024-10-10

**Authors:** Ignacio Mochales Cuesta, Sergio L. Jiménez-Sáiz, Adam L. Kelly, Álvaro Bustamante-Sánchez

**Affiliations:** 1 Faculty of Sports Sciences, Universidad Europea de Madrid, Tajo Street, s/n, 28670 Madrid, Spain; i.mochales.cuesta@gmail.com (I.M.C.); alvaro.bustamante@universidadeuropea.es (Á.B.-S.); 2 Sport Sciences Research Centre, Universidad Rey Juan Carlos, 28942 Madrid, Spain; 3 Research for Athlete and Youth Sport Development (RAYSD) Lab, Research Centre for Life and Sport Sciences (CLaSS), College of Life Sciences, Birmingham City University, Birmingham B5 5JU, UK; adam.kelly@bcu.ac.uk

**Keywords:** basketball, home advantage, player performance, crowd influence, travel influence

## Abstract

Background: This systematic review analyzes the factors that influence home advantage in basketball across various competitions in the United States and Europe. Methods: Through an investigation of English- and Spanish-language articles published in EBSCO, Scopus, Consensus, and Web of Science between 2010 and 2024 related to home advantage in basketball, 1682 articles were initially identified. After applying specific filters to ensure that only articles concerning National Basketball Association (NBA), Women’s National Basketball Association (WNBA), Euroleague, Spanish basketball, and European basketball were considered, 39 articles met the final requirements for in-depth analysis. Results: The studies analyzed in this review suggested that player performance, player position, and sleep influenced home advantage in competitions in Europe and the United States. Fan behavior had a bigger impact in European competitions, where teams from capital cities have a lower home advantage. In the United States, where teams must travel long distances to play, several studies indicated that teams traveling eastwards tend to perform more strongly than teams traveling westwards. Also of note is that, in many cases, COVID-19 pandemic restrictions reduced home advantage. Conclusions: This review identifies factors contributing to home advantage in basketball, compares competitions in different regions, and proposes ideas for future research such as a greater focus on women’s competitions, the impact of television, and the introduction of new performance indicators.

## 1. Introduction

Home advantage refers to the advantage that a team has when they play at home compared to their performance when they play away [1]. It exists when home teams win more than 50% of the games with a home and away schedule [2]. Home advantage in team sports is a well-known phenomenon that has been observed in various team sports in the United States such as basketball, baseball, and ice hockey [3] and in football in Europe [4].

While there are several articles about home advantage factors in football [4], there is still much to be learned about the factors that contribute to home advantage in basketball. This is a topic that has gained attention from researchers over the past decades [5,6], and it has been analyzed at professional [7,8,9] and amateur levels [5,10,11].

Home advantage in basketball has been analyzed in various studies that provide insights into team performance [11]. Home teams had a higher number of both defensive and offensive rebounds [11], better shooting percentages in field goals and free throws [12], and a higher number of steals and offensive blocks [11]. Another factor that affected home advantage is the influence of crowd support and familiarity with the court [2,13]. Before the game, the home team had a 62.0% winning probability, but if they were behind in the first quarter, the winning probability dropped to 44.2% [13]. Most of the reviewed studies focused only on one league or country. However, a comparison between different leagues and countries is still missing [14,15].

Although certain researchers have employed game-theoretic principles in relation to aspects of basketball strategy such as offensive game and away team’s rest effects [11,16,17], these have not yet been integrated into the overall view of home advantage. Furthermore, there is an emphasis on game statistics and offensive efficiency [11], which tend to be more related to the home advantage rather than the defensive strategy.

This systematic review focuses on the existing literature between 2010 and 2024, highlights the areas where more evidence is needed, provides an in-depth analysis of home advantage in basketball explaining different factors, and includes future research suggestions that may be used to investigate home advantage in basketball. This review includes a unique situation with the COVID-19 lockdown and the matches played without fans worldwide. There are a few articles that have analyzed how this situation impacted home advantage.

The goal of this review was to compare different competitions and understand if home advantage is influenced by the same factors in the United States and Europe and the differences between their competitions. The aim was to compare how home advantage is influenced in different professional basketball leagues around the world and understand the factors that are common to all of them and those that are specific to a competition.

We hypothesize that there are similarities between NBA and European competitions. Player performance and player position should be common to European and North American competitions, while travel distance plays a key role in the United States and not in Europe. Also, fan behavior is different in both regions and its influence should be bigger in European leagues.

## 2. Methodology

### 2.1. Design

This systematic review on home advantage in basketball followed the PRISMA (Preferred Reporting Elements for Systematic Review and Meta-Analysis) guidelines for conducting and reporting systematic reviews. The PRISMA guidelines ensure transparency and methodological rigor in the review process [18]. The review started with a comprehensive search of relevant literature using multiple databases and search terms related to home advantage in basketball.

### 2.2. Search Strategy

The databases used to find articles were EBSCO, Scopus, Consensus, and Web of Science.

A search strategy was implemented to identify documents on home advantage in basketball. The search strategy included the following keywords and phrases: basketball AND ((home OR away OR court OR ((professional AND leagues AND home)) AND advantage) OR ((crowd OR travel) AND influence) OR (away AND performance)). A total of 1682 unique articles were initially identified through the search strategy.

### 2.3. Inclusion Criteria

The inclusion and exclusion criteria were defined, focusing on articles published between 2010 and 2024 and articles written in English or Spanish. Only articles that focused on home advantage in basketball, specifically in men’s and women’s professional leagues including the NBA, WNBA, Euroleague, Spanish basketball, and European basketball, were included in this review. Any articles that did not meet these criteria were excluded from the review. This filter returned 42 articles.

**Figure 1:**
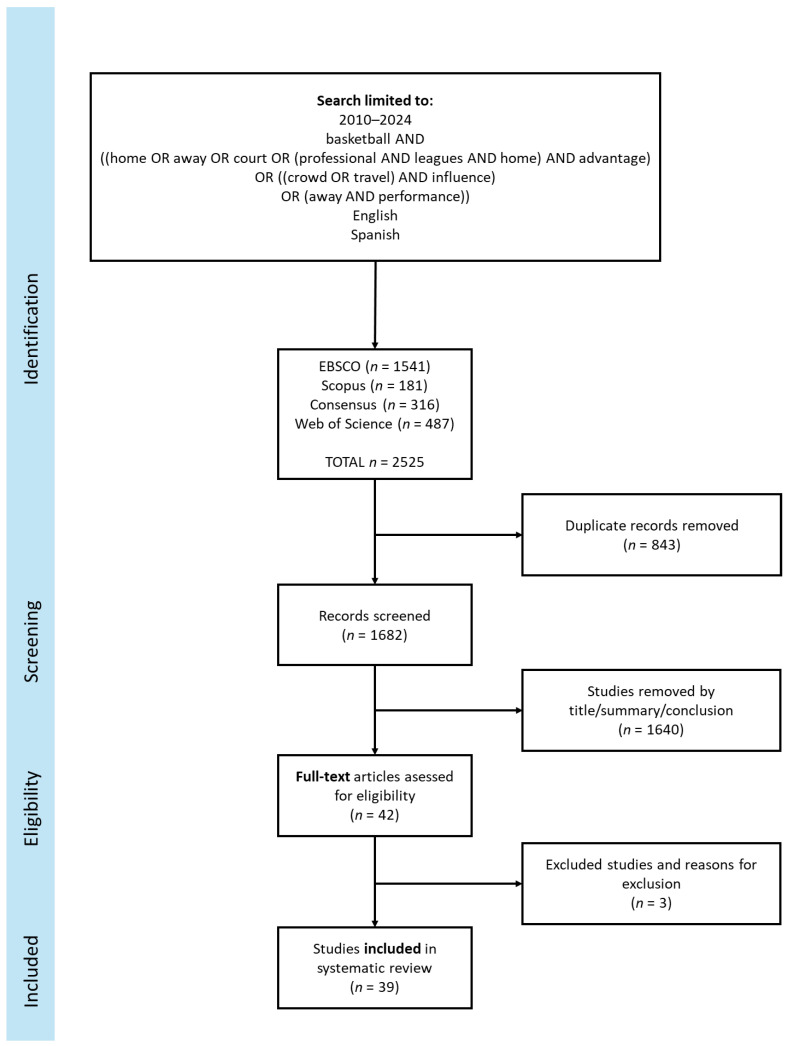
PRISMA study flow diagram.

The 42 articles remaining were read in detail and classified according to the competition or competitions that they covered. After reading them, three were excluded because they were not relevant as they were focusing only on one team. The 39 articles remaining were reviewed using a descriptive and analytical approach. The findings were structured in a detailed and organized manner, highlighting the key factors contributing to home advantage in basketball, analyzing the limitations of the reviewed studies, and discussing potential future research to improve the findings. Figure 1 illustrates the flowchart of the detailed process for selecting the studies.

### 2.4. Data Extraction

Relevant data were extracted, including information on study design, number of seasons, participant characteristics, statistical analysis methods, and findings.

The chronological analysis of the articles analyzed for this review proved that in recent years, home advantage in basketball has gained importance mainly because of the COVID-19 lockdown and the matches played without spectators or with a limited number of fans. In total, 56% of the articles reviewed were published in the last four years (2021–2024). Out of the 39 articles, 4 cover the Euroleague, 5 cover European leagues, 5 cover Spanish basketball, 24 cover the NBA and WNBA, and one article analyzes how sleep affects recovery and performance in basketball in multiple competitions [19]. Regarding the seasons covered, 44% of the articles cover three or less seasons while 36% of them cover ten or more.

This systematic review also provides suggestions for future research, such as considering more women’s competitions, more parties involved in the games, home and away fans, and new performance and physical metrics.

### 2.5. Data Analysis

Evaluating the risk of bias is a crucial step in the analysis of data in scientific research. The risk of bias in each article was evaluated based on guidelines provided by The Cochrane Collaboration. The following classifications were used: low risk, high risk, and unclear risk (either lack of information or uncertainty regarding the potential for bias).

**Figure 2:**
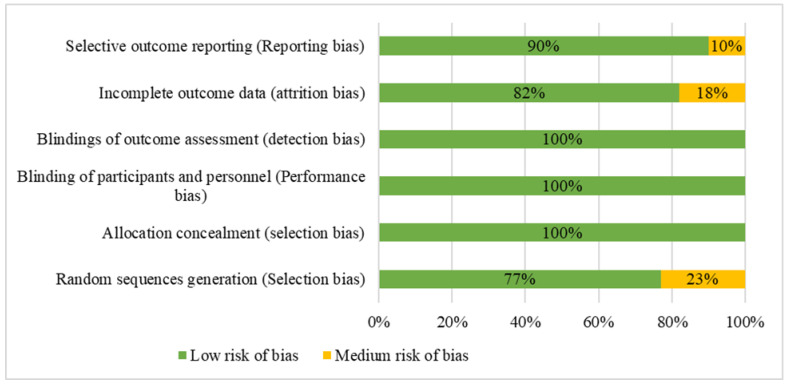
Risk of bias presented as percentages across all included studies.

Table 1 and Figure 2 show that most of the studies had a low risk of bias across most criteria. However, there were some areas, such as random sequence generation and incomplete outcome data, where a small percentage of studies exhibited a medium risk of bias. None of the identified studies had a high risk of bias. This result suggests an overall high quality in terms of methodological standards in the articles that were included in this review.

## 3. Results

The aim of this review was to compare the factors that influence home advantage in different competitions in the United States and Europe. The hypothesis was fulfilled because of factors such as player performance, player position, and sleep. However, crowd influence had a bigger impact in Europe, and travel distance only affected home advantage in the United States, as distances in Europe are smaller. This systematic review found only one article about women’s basketball.

A systematic review was conducted, and we ended up finding forty articles that focused on diverse aspects of home advantage in basketball. Specifically, the review analyzed four articles related to the Euroleague, five articles about European leagues, five articles analyzing Spanish basketball, twenty-four articles about the NBA, and one article focused on the WNBA. Additionally, there was a singular article that explored the impact of sleep on performance across multiple competitions [19].

The findings were categorized based on the competitions analyzed: Euroleague, European leagues, Spanish league, and NBA/WNBA. Despite similarities in the factors and discoveries explored in articles across these competitions, factors such as travel distance [51], crowd behavior [50], and the volume of articles for each competition warranted a division at the competition level.

Most of the studies in this review found that home teams have a higher winning percentage compared to away teams [26]. Home advantage in basketball can be attributed to varied factors including spectators’ influence [43], travel logistics [31], familiarity with the home court [29], and team performance [54]. Based on the analyzed literature, it is evident that home advantage is a consistent phenomenon in basketball across various leagues in the United States (NBA and WNBA) and Europe (Euroleague, Spain, Italy, Germany, Greece, Finland, etc.). Table 2 shows key information from the studies included in this review.

### 3.1. Home Advantage in the Euroleague

The four articles analyzing the Euroleague show unique aspects of home advantage and analyze how the absence of spectators because of COVID-19 restrictions affected home advantage [48]. The quality of the players [49,54], familiarity with the home court [29], and the influence of spectators [48] are significant factors contributing to home advantage in Euroleague basketball. Out of the four articles, two focused on one Euroleague season [49,54] and the two others covered one [29] and three seasons [48] analyzing the impact of COVID-19.

Sampaio et al. [54] focused on a specific season where home teams won 66% of their games and highlighted the influence of player positions on home advantage. They concluded that the guards from the home teams played with more confidence, while in the away teams, it was the forwards who played with more confidence. This is one of the few articles found that analyzed how the performance of specific player positions can impact the result of a game and how the home advantage and player position are linked.

In contrast, Pojskić et al. [49] adopted a broader perspective, comparing top-level leagues with lower-level competitions. They compared 118 regular season games, 48 playoff games, and 181 NLB–Adriatic games and found that home advantage exists more often in lower-level competitions at the result and game statistics level. In the regular season of the Euroleague, home teams won 66% of their games while, in the NLB–Adriatic league, teams won 67% of their games [49]. When the Euroleague teams played in the playoffs, home teams won 58% of their games [49]. In the NLB–Adriatic league, the starters from the home team scored more points on average than the starters of the away team [49]. Pojskić et al. [49] agree with Sampaio et al. [54], as they suggest that the home teams play more aggressively in defense than the away teams [49]. Pojskić et al. [49] and Sampaio et al. [54] analyzed how players playing in the same position had different performances when they played at home and away. In the playoffs, the home advantage was reduced to 58% because the quality of the teams was close [49]. When the level of competition increased, home advantage tended to be minimized as the impact of the home crowd was less relevant. The quality of the players could make home advantage disappear [49].

The comparison between top leagues and lower leagues also suggests that the organizers of the competitions influence home advantage when they create rules to play in similar courts, making sure that all of them have a similar layout. The organizers can also control the number of days of rest for the teams and they train and prepare the referees for the competition [49].

Two articles studied the crowd effect during COVID-19. Paulauskas et al. [48] analyzed how the pandemic changed tactics, player skills, and the traditional home advantage. Teams had to adapt to the absence of spectators and create new strategies to compensate for it [48]. On the other hand, Bourdas et al. [29] emphasized the importance of the home court in determining home advantage, even without fans. Spectators are important, but the familiar home-court environment plays a crucial role in home advantage [29].

The phenomenon of home advantage in the Euroleague is characterized by multiple factors. Primarily, player performance is influenced by the venue [29]. Sampaio et al. [54] and Pojskić et al. [49] agreed that players exhibit various levels of confidence when they play at home or away. Home advantage was more pronounced when the quality of the teams and the players was low [49]. An analysis of seasons played in the presence and absence of spectators reveals significant changes in team strategies and player tactics, which are needed to compensate for the absence of home advantage [48]. Even in the absence of spectators, the familiar facilities of the home venue create an advantage for the home team [29].

### 3.2. Home Advantage in National European Basketball Leagues

European basketball has been reviewed in recent years with complete analyses of a good range of leagues of distinct levels and several seasons [33]. While some authors focused on the influence of COVID-19 and how teams performed with and without spectators [20,22,33], others focused on the geographical aspect of home advantage and how the profile and the behavior of the spectators affected the performance of home teams [37,50]. Three out of the five articles analyzed compared more than 16 seasons of European leagues to analyze how the presence of fans affected home advantage [20,22,33].

De Angelis and Reade [33] analyzed the impact of COVID-19 on 10 European leagues: Spain, Russia, Turkey, France, Italy, Germany, Greece, the Adriatic League, Israel, and Lithuania. They explored the impact of the COVID-19 pandemic on European basketball. With 26,675 pre-pandemic and 1026 post-pandemic matches analyzed, their research revealed a 5.1% reduction in home-winning probability in top European basketball leagues in games played without spectators in 2020 [33]. It is important to note that this reduction persisted when spectators returned to the arenas [33]. In eight leagues, home advantage decreased after COVID-19, with Lega Basket Seria A in Italy with an 11.3% decrease [33]. There were two leagues where home advantage increased after COVID-19: Greece and Lithuania [33].

Alonso Pérez-Chao et al. [20] agreed with De Angelis and Reade [33] and found that European teams exhibited higher home advantage in pre-pandemic matches [20]. They collected data from Spain, Germany, Italy, Greece, and Israel and discovered that team ability level had a greater influence on game outcomes than home advantage [20]. Alonso Pérez-Chao et al. [20] agreed with the analysis that Pojskic et al. [49] prepared about the Euroleague teams and discovered that home advantage decreases when team ability increases. Alonso Pérez-Chao et al. [21] extended their analysis and compared home advantage with spectators’ attendance and found a correlation between them. In the period of 2005–2021, matches with fans increased the home advantage in the five European leagues analyzed [50].

Gómez Ruano & Pollard [37] and Pollard & Gómez Ruano [50] focused on the geographical aspect of home advantage in European basketball. Their research revealed that capital city teams exhibited lower home advantage [37]. Gómez Ruano and Pollard [37] believe that the reason behind this is the characteristics of the inhabitants of capital cities, where there is less sense of territorial protection. They also analyzed the Balkan region and discovered that teams in the Balkan region protected their territory more intensely, influencing their performance in games [50]. With a 72.8% home advantage in Bosnia–Herzegovina and a 70.3% home advantage in Croatia, Alonso Pérez-Chao et al. [21] agreed with Pollard and Gómez Ruano [50] and considered that spectators can clearly influence the outcome of games, and their behavior is particularly important in increasing or decreasing home advantage. If spectators feel that their team represents their values and feel connected to their team, they will show their support and help to increase home advantage [50].

### 3.3. Home Advantage in Spanish Basketball

The Asociación de Clubs de Baloncesto (ACB) is the top professional basketball league in Spain. It is one of the top three leagues in the world after the NBA and Euroleague and the top national league in Europe. The league features 18 teams competing in a double round-robin format with each team playing 34 games. The bottom two teams face relegation and the top eight teams enter the playoffs. Three studies focused on four specific seasons of the ACB [12,24,35], and two studies analyzed several seasons of the ACB comparing the league with other European Leagues [24,33].

The study conducted by Navarro Barragán et al. [12] analyzed the critical moments of 30 games of the ACB in the 2007–2008 season. They focused on player performance and discovered that, when playing at home, defensive rebounds, free throws, and two-point throws were key elements that influenced home advantage [12]. Sampaio et al. [54] agreed with Navarro Barragán et al. [12] and Pojskić et al. [49]: home teams played better in defense than away teams. Home teams had more defensive rebounds than away teams and that means that they had more ball possession and more opportunities to score [12]. Two stats were very relevant in understanding the home advantage in Spain: home teams made more free throws than away teams, and away teams missed more two-point throws than home teams [12].

García Rubio et al. [35] agreed with Navarro Barragán et al. [12], Sampaio et al. [54], and Pojskić et al. [49] and reinforced the superiority of home teams in defensive rebounds. Away teams changed their strategy and played away from the basket proving how spectators and territoriality affect the psychology of the players and team performance [35]. The study conducted in 2023 by Alonso Pérez-Chao et al. [21] analyzed the performance of home and away teams in the top U18 basketball competitions in Spain. They discovered that, at lower levels, external physical demand influence is similar when teams play at home and away, so this is not a factor that creates a home advantage for the teams [24].

De Angelis and Reade [33] analyzed the impact of COVID-19 on 10 European leagues including Spain. In 3927 matches analyzed between 2004 and 2021, their research revealed an 8.3% reduction in home-winning probability in Spain in games played without spectators in 2020 [33]. This reduction persisted when spectators returned to the arenas which suggested that familiarity with the home venue was not a factor that could explain home advantage in Spain [33].

Alonso Pérez-Chao et al. [21] collected data from five European leagues including Spain and discovered that team ability level had a greater influence on game outcomes than home advantage [24]. Teams with high ability levels won 56.5% of their matches with fans and 50.9% of their matches without fans while low ability teams won 68.2% of their matches with fans and 61.4% of their matches without fans [24], proving that home advantage decreases when team ability increases [49]. Alonso Pérez-Chao et al. [23] compared male and female basketball and concluded that home advantage is smaller for female teams with a more even distribution of wins when they played at home and away [23]. In their analysis of spectator’s influence on women’s basketball in Spain, Alonso Pérez-Chao et al. [23] concluded that home advantage increased without fans during the pandemic compared to pre-pandemic games [23].

### 3.4. Home Advantage in NBA and WNBA

The NBA and the WNBA are the most popular basketball leagues in the world for men and women. The systematic review found 25 articles that analyzed these competitions over several seasons. Out of the 25 articles, only one explored home advantage at women’s level at the WNBA [47]. Orton et al. [47] were the only authors in this systematic review that analyzed how home advantage affects professional women’s basketball.

Three factors were identified in the 25 articles that allowed for grouping them into various categories: spectators’ influence, player performance, and travel logistics.

The eight articles that analyzed crowd influence in home advantage made similar findings. Barreira and Morgado [26] introduced a natural experiment comparing two teams that played at the same venue when they were the home team and played against each other four times per season. The likelihood of home teams winning suggested a notable biased crowd effect [28]. Spectators’ behavior during the games influenced the home team’s probability of winning [28].

Barreira and Morgado [26] analyzed 76 seasons of the NBA between 1946 and 2022 and noted that home advantage in the NBA remained consistent with a percentage of around 65%, but with a significant decrease since 1965 [26]. The 2019–2020 season, marked by the absence of spectators due to COVID-19, contributed to more balanced matches [26]. García Rubio et al. [36] indicated a 59.6% home advantage in the NBA between the 2006–2007 and 2012–2013 seasons. Teams from larger cities experienced a lesser advantage, and factors like large distances between teams, arena capacity, and crowd density were also relevant [36]. The study emphasized how the presence of spectators affected revenue and team performance [36].

Leota et al. [43] agreed with Barreira and Morgado [26] in their analysis of the 2020–2021 season of the NBA. Leota et al. [37] noted that empty arenas eliminated home advantage. However, in games with crowds, a substantial home advantage returned [43]. Price and Yan [52] also analyzed the 2019–2020 season and its previous seasons. They found that away teams performed better in the absence of fans [52]. Away teams experienced an improvement during the COVID-19 season, and their performance was affected by the influence of travel, fan pressure, and unfamiliar courts [52].

Böheim et al. [27] found a relationship between the number of spectators and the performance of the players. Their study explored the effects of audience size on free throw success in the first half of the matches [27]. This effect was more pronounced for less skilled players, highlighting the psychological pressure created by large crowds and how it affected player performance [27]. Orton et al. [47] made similar findings as Böheim et al. [27] about the impact of the number of spectators on the performance of the teams. Orton et al. [47] showed how the number of spectators affected the offensive and defensive performance of home teams, which received fewer personal fouls and made more free throw attempts [47].

Several authors have analyzed how home advantage affected the performance of the players of home and away teams. Lu et al. [45] identified different key factors for wins during the COVID-19 pandemic, such as free throws, three-point throws, defensive rebounds, assists, steals, fouls, and opponent quality. Kozy James [42] analyzed the 2008–2009 season of the NBA and discovered how the home advantage affected the two-point shooting percentages for visiting teams. The conclusion suggested a change in the strategy of away teams as they should increase their two-point attempts to leverage the disadvantage of playing away [42]. Bustamante-Sánchez et al. [30] agreed with Lu et al. [45] and Kozy James [42] in their study of the 2019–2020 season of the NBA and demonstrated that home teams exhibited superior performance in assists, rebounds, and shooting percentage than away teams. COVID-19 proved how home advantage was impacted not only by home fans but also by the game location [30].

Cheng [32] compared the performance of home and away teams in the 2013–2018 seasons of the NBA and discovered that teams scored, on average, 2.3 points more when they played at home. Home advantage remains even if teams can control factors such as team strength, rest, and travel [32]. Cheng [32] agreed with Ribeiro et al. [53] and observed that teams scored more at home, with an average increase of 0.13 points per minute. Home advantage appeared to reduce gradually over seasons [53]. On the other side, Graham et al. [38] discovered, in their study, that home advantage did not exist in decisive games, where home teams won less than away teams (63% vs. 66%). Regarding performance, defensive rebounds and steals increased in game five of the NBA playoffs when the score was 3–1 [38].

García Rubio et al. [36] agreed with Kotecki [41] and considered that increasing attendance improved revenue and the home team’s chances of winning. His analysis of the 2008–2011 seasons of the NBA revealed that home teams had better field-goal percentages, and overall statistics, compared to away teams [41]. Böheim et al. [27] and Orton et al. [47] found that there was a relationship between the number of spectators and performance revealing a negative impact on free throws for men’s and women’s basketball. The number of fans negatively affected the performance of less skilled players, proving how spectators created psychological pressure on players [27]. Home teams exhibited superior offensive and defensive efficiency thanks to the impact of large crowds [47]. Ganz and Allsop [34] concluded that fans influence the home team performance with a 1.69-point increase per game and a difference of 5.7 home wins per season when matches are played with spectators [34].

Harris and Roebber [39] introduced artificial intelligence into the analysis, revealing that the percentage of two-point shots was better for the home team, and the away team was more successful with three-point shots. They also concluded that referee bias played a role in home advantage [39].

Singh Abrol et al. [55] analyzed 60 seasons of the NBA and discovered how psychological factors affected the performance of both home and away teams. Teams in good form tended to win games at home and away, with home teams winning more than 50% of their games showing that home advantage existed during 60 seasons of the NBA [55].

Over the years, travel related to sports has become more convenient, faster, and comfortable [55]. A few articles in this systematic review analyzed the link between home advantage and the fatigue produced by the journeys of the away teams.

Pradhan et al. [51] analyzed the effects of travel on NBA playoff performances. Teams that traveled eastward had more assists and higher field-goal percentages and committed more fouls than teams that traveled westward [51]. The circadian rhythm played a crucial role in regulating various physiological and behavioral processes and was a crucial factor that impacted team performance [51]. Leota et al. [44] agreed with Pradhan et al. [51] in their analysis of eastward jet lag in the NBA during the 2011–2021 seasons. While eastward jet lag negatively impacted away teams, westward jet lag did not show any significant impact [44]. Charest et al. [31] made similar discoveries in their analysis about the link between travel direction and performance in the NBA. They suggested that teams should create recovery strategies to compensate for the accumulation of travel fatigue and circadian desynchronization [31]. Huyghe et al. [40] analyzed how air travel influenced the health and performance of away teams. Different time zone transitions increased injury risk, reduced performance, and disrupted the circadian rhythm of the players [40].

McHill and Chinoy [46] disagreed with the previous authors and considered that traveling westward affected player performance. In their analysis, they concluded that teams traveling westward showed decreased winning percentages, shooting accuracy, and effort, and increased points allowed [46].

Fatigue was also a key factor for Ashman et al. [25] and Zhang et al. [56]. Fatigue and other factors influencing home advantage played a surprising role in wagering markets [25]. Accumulated fatigue has an impact on home advantage and teams should manage their schedules to make sure that it has no effect on their performance [56]. Too much rest time could be negative for the performance of the away team [56].

Ochoa-Lácar et al. [19] analyzed several articles about how sleep affected the performance of teams. They concluded that sleep was an important factor and could provoke injuries [19]. Trips, type of training, and schedule of training affected the rest of the players and should be considered when teams create recovery strategies [19].

## 4. Limitations and Recommendations for Future Research

While this systematic review provided valuable insights about multiple competitions and factors, there were some limitations found in the reviewed studies. Future research should address these limitations to ensure a full understanding of home advantage in basketball. As the understanding of home advantage continues to evolve, new investigations could dig into new aspects that could affect the outcome of the games.

Future studies could investigate how the home crowd influences the performance of home and away teams. This could include the psychological effect of spectators on players and the strategies used by teams to leverage the effect of the home crowd. A complete analysis should also include how the percentage of home and away fans and their density affect the outcome of the games.

The potential bias of referees should be investigated to understand how it affects home advantage. Future research could focus on analyzing referee behavior in different leagues, their stats with different teams, the percentage of controversial decisions for home and away teams, and how the crowd influences their decisions in critical moments of the games.

While there is a sizable number of documents about home advantage in men’s basketball, this systematic review found only two articles about women’s basketball. A good comparison of the factors that affect home advantage in men’s basketball should also be considered in women’s basketball to obtain a full overview of home advantage in basketball.

Research exploring the impact of specific game situations, such as score differentials, game time, results in other games, number of fouls per team, and time left could provide new insights about home advantage.

In-depth interviews or surveys with players, coaches, team staff, referees, and spectators could add value and give new insights into how they perceive and adapt to the challenges of playing at home or away. Understanding the psychological side of the parties involved in basketball can help to understand the home advantage.

Advanced performance metrics can offer a better understanding of home advantage. Sensors and devices can track player data to know more about their performance. These metrics could offer distance covered, speed, and acceleration of players in home and away matches that can be connected to their performance. Physical sensors used by players, coaches, and referees could provide useful data to understand how the performance affects home advantage.

The impact of television can also be analyzed to check if the players behave in a unique way when they know that the cameras are recording all their moves and actions. Players could play more aggressively if they knew that only the referees could suspend them, and if all the plays would not be reviewed by checking the images on television.

This systematic review has found several studies about home advantage in basketball leagues in Europe and the United States. Future research could analyze how home fans influence international tournaments such as the FIBA World Cup, European Championships, Olympic Games, or the Final Four of the Euroleague. Fan support could impact the outcome of the matches played in these types of tournaments where a win can mean that a team qualifies for the next round.

The suggested research areas offer valuable opportunities to deepen the understanding of home advantage in basketball. By addressing these areas, future research can provide new insights and explain home advantage in basketball.

## 5. Conclusions

The goal of this systematic review was to compare the factors that influence home advantage in different basketball competitions. Different leagues and tournaments across the United States and Europe were analyzed in this review. The main factors that affect home advantage in basketball are player performance, the behavior of spectators, familiarity with the home court, geography, travel distance, and sleep. The performance of the players and their position is different when they play at home and away, and home advantage tends to disappear when the level of the league increases. The fans play a key role, and they can influence the outcome of games, but teams from capital cities exhibit lower home advantage. Travel distance and the direction of the travel are key factors in the NBA with teams flying eastward having a better performance than teams that traveled westward. There are a few studies that analyze how good sleep improves the performance of the players. It is interesting how women are affected by the same factors as men regarding home advantage: home teams exhibited superior offensive and defensive efficiency thanks to the impact of large crowds.

Three factors are common in basketball competitions in Europe and the United States: player performance, player position, and sleep. The main factor that influences home advantage in basketball that has been identified in this systematic review is player performance, with teams exhibiting similar stats when they play at home and other stats when they play away. Player position has also been identified as a key factor with guards and forwards playing with different confidence levels at home and away. Sleep is a common factor in all competitions, as it affects performance and can provoke injuries. The influence of the crowd clearly influences the outcome of the games with differences between the United States and Europe, where the behavior of the fans has a bigger impact on the outcome of the basketball games.

In Europe, teams from capital cities have lower home advantage, while in the United States, teams must travel long distances to play, and several studies have probed that teams traveling eastwards have a better performance than teams traveling westwards.

Player performance, player position, and sleep are common factors in the United States and in Europe. Crowd influence has a bigger influence on European basketball. The type of city influences home advantage in European basketball, while travel fatigue is a key factor in the United States.

## Figures and Tables

**Table 1:** Risk of bias. Study quality assessment.

Studies	Random Sequences Generation (Selection Bias)	Allocation Concealment (Selection Bias)	Blinding of Participants and Personnel (Performance Bias)	Blinding of Outcome Assessment (Detection Bias)	Incomplete Outcome Data (Attrition Bias)	Selective Outcome Reporting (Reporting Bias)
Alonso Pérez-Chao et al. [20] (2022)	+	+	+	+	+	+
Alonso Pérez-Chao et al. [21] (2023)	?	+	+	+	+	+
Alonso Pérez-Chao et al. [22] (2024)	+	+	+	+	+	+
Alonso Pérez-Chao et al. [23] (2024)	+	+	+	+	+	+
Alonso Pérez-Chao et al. [24] (2024)	+	+	+	+	+	+
Ashman et al. [25] (2010)	+	+	+	+	+	+
Barreira & Morgado [26] (2023)	+	+	+	+	?	?
Böheim et al. [27] (2019)	+	+	+	+	+	+
Boudreaux et al. [28] (2017)	+	+	+	+	+	+
Bourdas et al. [29] (2022)	?	+	+	+	+	+
Bustamante-Sánchez et al. [30] (2022)	?	+	+	+	+	+
Charest et al. [31] (2021)	+	+	+	+	+	+
Cheng [32] (2019)	+	+	+	+	+	+
De Angelis & Reade [33] (2022)	+	+	+	+	+	+
Ganz & Allsop [34] (2024)	+	+	+	+	+	+
García Rubio et al. [35] (2014)	+	+	+	+	+	+
García Rubio et al. [36] (2015)	+	+	+	+	+	+
Gómez Ruano & Pollard [37] (2011)	+	+	+	+	+	+
Graham et al. [38] (2022)	+	+	+	+	?	?
Harris & Roebber [39] (2019)	+	+	+	+	?	?
Huyghe et al. [40] (2018)	+	+	+	+	+	+
Kotecki [41] (2014)	+	+	+	+	+	+
Kozy [42] (2011)	+	+	+	+	+	+
Leota et al. [43] (2022)	+	+	+	+	+	+
Leota et al. [44] (2022)	?	+	+	+	?	+
Lu et al. [45] (2022)	?	+	+	+	?	+
McHill & Chinoy [46] (2020)	?	+	+	+	+	+
Navarro Barragán et al. [12] (2012)	+	+	+	+	+	+
Ochoa-Lácar et al. [19] (2022)	+	+	+	+	+	+
Orton et al. [47] (2022)	+	+	+	+	+	+
Paulauskas et al. [48] (2022)	?	+	+	+	+	+
Pojskic et al. [49] (2011)	+	+	+	+	+	+
Pollard & Gómez Ruano [50] (2013)	+	+	+	+	+	+
Pradhan et al. [51] (2022)	+	+	+	+	+	+
Price & Yan [52] (2021)	?	+	+	+	+	+
Ribeiro et al. [53] (2016)	+	+	+	+	+	+
Sampaio et al. [54] (2013)	+	+	+	+	+	+
Singh Abrol et al. [55] (2021)	+	+	+	+	?	?
Zhang et al. [56] (2023)	?	+	+	+	?	+

+ indicates low risk and ? indicates unclear risk.

**Table 2:** Evidence table.

Study	Sample	Intervention Protocol	Outcome Measurement	Conclusions
Alonso Pérez-Chao et al. [20] (2022)	All games of 10 European leagues between the 2005–2006 and 2020–2021 seasons	Analysis of home vs. away performance and influence of spectators	Home win percentage and total games played	European teams exhibited higher home advantage and home win percentages pre-pandemic compared to the post-pandemic period. Team ability level had a greater influence on game outcomes than home advantage.
Alonso Pérez-Chao et al. [21] (2023)	16 games of the U18 Spanish basketball league during the 2019–2020 and 2020–2021 seasons	Analysis of external peak demands	Data obtained with accelerometer, magnetometer, and gyroscope	External physical demands are consistent regardless of venue. Higher physical demands were observed in home games, suggesting a minor effect of playing venue.
Alonso Pérez-Chao et al. [22] (2024)	All Spanish top-division male and female games between the 2010–2011 and 2022–2023 seasons	Analysis of home vs. away performance comparing male and female competitions	Home win percentage	Males had a bigger home advantage than females in both higher- and lower-ability teams.
Alonso Pérez-Chao et al. [23] (2024)	4780 games of women’s first division in Spain between the 2010–2011 and 2022–2023 seasons	Analysis of home vs. away performance	Home win percentage and performance-related variables	There were no home advantage differences when fans were absent during the COVID-19 lockdown.
Alonso Pérez-Chao et al. [24] (2024)	All games of 10 European leagues between the 2005–2006 and 2020–2021 seasons	Analysis of the influence of spectators, geographical location, and home vs. away performance	Home win percentage and performance-related variables	Home advantage was significantly associated with fan attendance. Matches with fans produced greater home advantage and home win percentages.
Ashman et al. [25] (2010)	All regular season NBA games between the 1990–1991 and 2008–2009 seasons	Analysis of home vs. away performance and rest days	Home win percentage and rest time	The betting market consistently mispriced games, especially for home underdogs revealing market inefficiencies in assessing team performance.
Barreira & Morgado [26] (2023)	4259 NBA playoff games between the 1946–1947 and 2021–2022 seasons	Analysis of home vs. away performance	Home win percentage and performance-related variables	Home advantage in the NBA remained consistent at around 65%, but a significant decrease was observed after 1965. During the 2019–2020 season, the absence of the public due to COVID-19 contributed to more balanced matches.
Böheim et al. [27] (2019)	10,578 NBA games between the 2007–2008 and 2015–2016 seasons	Analysis of home vs. away performance and influence of spectators	Home win percentage, attendance, crowd density, and performance-related variables	Larger crowds lead to decreased free throw success, particularly in the first half and when trailing. The negative effect is more pronounced for relatively worse players.
Boudreaux et al. [28] (2017)	59 NBA regular season games between the Los Angeles Lakers and Los Angeles Clippers between the 1999–2000 and 2013–2014 seasons	Analysis of home vs. away performance and influence of spectators	Home win percentage and attendance	Significant increases in home team win likelihood, suggesting biased crowd effects, are notable.
Bourdas et al. [29] (2022)	224 Euroleague games during the 2019–2020 and 2020–2021 seasons	Analysis of the influence of spectators	Home win percentage	Home advantage impacts winning frequency and reduces turnovers for Euroleague home teams.
Bustamante-Sánchez et al. [30] (2022)	906 NBA games from the 2019–2020 season	Analysis of home vs. away performance	Home win percentage and performance-related variables	Home teams demonstrate superior performances in assists, rebounds, and shooting percentages.
Charest et al. [31] (2021)	All home and away NBA games in two different cities between the 2013–2014 and 2020–2021 seasons	Analysis of home vs. away performance, travel distance, and travel direction	Home win percentage, travel distance, and direction of travel time	The accumulation of travel fatigue and chronic circadian desynchronization can disturb sleep and recovery. Tailored sleep and recovery strategies need to be developed throughout the season.
Cheng [32] (2019)	All NBA games between the 2013–2014 and 2017–2018 seasons	Analysis of home vs. away performance	Home win percentage and performance-related variables	An additional 2.3 points were scored when playing at home. The home advantage effect remains even after controlling for factors such as team strength, rest, and travel.
De Angelis & Reade [33] (2022)	27,691 games of 10 European leagues between the 2004–2005 and 2020–2021 seasons	Analysis of the influence of spectators	Home win percentage	There was a 5% reduction in home-winning probability in top European basketball leagues during games played without spectators in 2020. This reduction persists over time.
Ganz & Allsop [34] (2024)	All NBA matches between the 2014–2015 and 2021–2022 seasons	Analysis of home vs. away performance and influence of spectators	Home win percentage, attendance, crowd density, and performance-related variables	Games with fans provide a home-court advantage, increasing the home team’s score by 1.69 points compared to games played without fans.
García Rubio et al. [35] (2014)	306 games of ACB league from the 2007–2008 season	Analysis of home vs. away performance	Home win percentage and performance-related variables	Home teams have better stats in defensive rebounds and achieving successful two-point field goals through effective assists. Away teams focus on plays away from the basket. Crowd influence, territoriality, and psychological factors impact team performance.
García Rubio et al. [36] (2015)	All NBA games between the 2006–2007 and 2012–2013 seasons	Analysis of home vs. away performance and influence of spectators	Home win percentage, attendance, crowd density, and performance-related variables	Home-court advantage in the NBA during the 2006–2007 and 2012–2013 seasons was 59.6%. Teams from large cities experience this advantage less. Factors such as long distances between teams, stadium capacity, and crowd density also influence the home-court advantage.
Gómez Ruano & Pollard [37] (2011)	7432 games of seven European leagues between the 2002–2003 and 2008–2009 seasons	Analysis of the influence of spectators and geographical location	Home win percentage	Clear evidence of home advantage exists in European basketball leagues. Capital city teams generally exhibit lower home advantage.
Graham et al. [38] (2022)	All NBA conference finals and finals between the 1979–1980 and 2018–2019 seasons	Analysis of home vs. away performance	Home win percentage and performance-related variables	Home teams win slightly less (63% vs. 66%) in decisive games. Defensive rebounds and steals increase in game 5 when trailing 3–1.
Harris & Roebber [39] (2019)	All NBA regular games between the 1983–1984 and 2017–2018 seasons	Analysis of home vs. away performance and influence of spectators	Home win percentage, attendance, crowd density, and performance-related variables	Two-point shots are favored at home games and three-point shots are favored at away games. Referee bias and crowd influence may play a role in the observed home advantage.
Huyghe et al. [40] (2018)	36 articles about travel requirements	Analysis of the influence of schedule and travel requirements	Travel distance, rest time, and performance-related variables	There is a need for effective strategies addressing sleep and travel fatigue in NBA players, promoting equity across teams.
Kotecki [41] (2014)	All NBA matches of four NBA teams between the 2008–2009 and 2011–2012 seasons	Analysis of home vs. away performance	Home win percentage and performance-related variables	Home teams consistently have better records, field-goal percentages, and statistics compared to away teams. Increasing attendance not only increases revenue but also improves the team’s chances of winning.
Kozy [42] (2011)	All NBA games between the 1999–2000 and 2009–2010 seasons	Analysis of home vs. away performance	Home win percentage and performance-related variables	Home-court advantage disproportionately affects two-point shooting percentages for visiting teams, suggesting that they should increase two-point attempts.
Leota et al. [43] (2022)	1080 NBA games from the 2020–2021 season	Analysis of home vs. away performance and influence of spectators	Home win percentage and performance-related variables	Empty arenas during the 2020–2021 NBA season eliminated home advantage, but in games with crowds, a substantial home advantage returned. Crowd presence influenced home team rebounding.
Leota et al. [44] (2022)	11,481 NBA games between the 2011–2012 and 2020–2021 seasons	Analysis of home vs. away performance and travel direction	Home win percentage, direction of travel, and performance-related variables	The analysis of 11,481 NBA games from 2011–2012 to 2020–2021 supports the idea that eastward jet lag negatively affects home teams. It produces fewer wins and impaired performance. Westward jet lag shows no significant impact.
Lu et al. [45] (2022)	1549 NBA matches during the 2019–2020 and 2020–2021 seasons	Analysis of home vs. away performance and influence of spectators	Home win percentage and performance-related variables	COVID-19 altered home advantage dynamics in the NBA. Key factors for wins included free throws, three-pointers, defensive rebounds, assists, steals, fouls, and opponent quality.
McHill & Chinoy [46] (2020)	1364 NBA games from the 2019–2020 season	Analysis of travel and player performance	Home win percentage, travel distance, and performance-related variables	Teams traveling westward showed decreased winning percentages, shooting accuracy, and effort, and increased points allowed. COVID-19 reduced traditional home-court advantages.
Navarro Barragán et al. [12] (2012)	30 games of ACB league from the 2007–2008 season	Analysis of home vs. away performance	Home win percentage and performance-related variables	Winning teams playing at home had better defensive rebounds and successful free throws compared to losing teams. Conversely, when playing away, winning teams had more missed two-point field goals than losing teams.
Ochoa-Lácar et al. [19] (2022)	28 studies about sleep and player performance	Analysis of the influence of sleep	Home win percentage and performance-related variables	The scientific literature emphasizes the crucial role of sleep and circadian rhythms in basketball performance. Sleep is a fundamental aspect of athlete recovery, with implications for both performance and health.
Orton et al. [47] (2022)	All matches of 10 WNBA teams between the 2015–2016 and 2018–2019 seasons	Analysis of home vs. away performance and referee bias	Home win percentage, attendance, and crowd density	Home teams have greater offensive and defensive efficiency. Crowd density’s impact on referee bias is inconclusive. Larger crowds positively affect performance efficiency.
Paulauskas et al. [48] (2022)	492 Euroleague games during the 2018–2021 season of the Euroleague	Analysis of home vs. away performance and influence of spectators	Home win percentage and performance-related variables	The COVID-19 pandemic altered basketball games diminishing performance and the home advantage. Changes in tactics, player abilities, and spectator absence contributed to these effects.
Pojskic et al. [49] (2011)	118 Euroleague regular season games, 181 NLB–Adriatic league games, and 48 Euroleague top 16 games from the 2008–2009 season	Analysis of home vs. away performance	Home win percentage and performance-related variables	Home advantage is more pronounced in lower-quality basketball competitions, influencing win–loss records and game-related statistics. At higher levels, home-court advantage becomes less crucial.
Pollard & Gómez Ruano [50] (2013)	17,099 games of 35 leagues in Europe between the 2009–2010 and 2011–2012 seasons	Analysis of the influence of spectators and geographical location	Home win percentage	In the Balkan region, teams seem to protect their territory more and it affects how well they perform in games.
Pradhan et al. [51] (2022)	499 NBA games between the 2013–2014 and 2018–2019 seasons	Analysis of home vs. away performance and travel direction	Home win percentage, direction of travel, and performance-related variables	Teams traveling east tend to have more assists, commit more fouls, and score higher field-goal percentages. Circadian misalignment impacts team performance. Teams could use strategies to cope with these effects.
Price & Yan [52] (2021)	486 NBA playoff games between the 2017–2018 and 2019–2020 seasons	Analysis of home vs. away performance and influence of spectators	Home win percentage and performance-related variables	Teams perform better in the absence of fans. Removing home advantage in 2020 did not show regression in home team performance, but away teams experienced improvement. Travel, fan pressure, and unfamiliar courts contribute to the negative effects on away teams and influence home advantage.
Ribeiro et al. [53] (2016)	16,133 NBA games between the 2001–2002 and 2013–2014 seasons	Analysis of home vs. away performance	Home win percentage and performance-related variables	Teams score slightly more at home, with an average increase of 0.13 points per minute. This home advantage appears to diminish gradually over seasons. Home advantage is mostly accumulated at the beginning of matches.
Sampaio et al. [54] (2013)	225 games during the 2004–2005 season of the Euroleague	Analysis of home vs. away performance	Home win percentage and performance-related variables	Players from the home team who play as guards acted confidently and players from the away team who play as forwards showed they were strong. Coaches can use this information to pick players and get the team ready.
Singh Abrol et al. [55] (2021)	All NBA games between the 1950–1951 and 2009–2010 seasons	Analysis of home vs. away performance	Home win percentage and performance-related variables	Teams perform better at home, supporting psychological factors.
Zhang et al. [56] (2023)	1214 NBA games from the 2021–2022 season	Analysis of home vs. away performance, travel distance, and rest days	Home win percentage, travel distance, and rest time	Schedule congestion affects NBA home advantage. Accumulated fatigue has a long impact on home advantage.
